# Why Do Patients Opt for the Emergency Department over Other Care Choices? A Multi-Hospital Analysis

**DOI:** 10.5811/westjem.18647

**Published:** 2024-09-25

**Authors:** Charles W. Stube, Alexander S. Ljungberg, Jason A. Borton, Kunal Chadha, Kyle J. Kelleran, E. Brooke Lerner

**Affiliations:** * University at Buffalo, Department of Emergency Medicine, Buffalo, New York; † University at Buffalo, Department of Pediatrics, Buffalo, New York; ° Posthumous Authorship

**Keywords:** emergency department, urgent care center, primary care physician, telehealth, hospital utilization

## Abstract

**Introduction:**

There are several options for receiving acute care besides emergency departments (ED), such as primary care physician (PCP) offices, urgent care centers (UCC), and telehealth services. It is unknown whether these alternative modes of care have decreased the number of ED visits for patients or whether they are considered before visiting the ED. A comprehensive study considering all potential methods of care is needed to address the evolving landscape of healthcare. Our goal was to identify any factors or barriers that may have influenced a patient’s choice to visit the ED as opposed to a UCC, PCP, another local ED, or use telehealth services.

**Methods:**

We surveyed ED patients between three hospital sites in the greater Buffalo, NY, area. The survey consisted of questions regarding the patients’ reasons and rationale for choosing the ED over the alternative care options. The study also involved a health record review of the patients’ diagnoses, tests/procedures, consults, and final disposition after completion of the survey.

**Results:**

Of the 590 patients consented and surveyed, 152 (25.7%) considered seeking care at a UCC, 18 (3.1%) considered telehealth services, and 146 (24.7%) attempted to contact their PCP. On the recommendation of their PCP, patients presented to the ED 110 (20.7%) times and on the recommendation of the clinician at the UCC 54 (9.2%) times. Patients’ perceived seriousness of their condition was the most common reason for their selected mode of transport to the ED and reason for choosing the ED as opposed to alternative care sites (PCP, UCC, telehealth). Based on criteria for an avoidable ED visit, 83 (14.1%) ED patients met these criteria.

**Conclusion:**

Individuals prioritize the perceived severity of their condition when deciding where to seek emergency care. While some considered alternatives (PCP, UCC, telehealth services), uncertainties about their condition and recommendations from other clinicians led many to opt for ED care. Our findings suggest a potential gap in understanding the severity of symptoms and determining the most suitable place to seek medical care for these particular conditions.

Population Health Research Capsule What do we already know about this issue?

*Traditionally, patients opt for the emergency department (ED) over other medical options due to many factors including access limitations, perceived urgency, convenience, and recommendations from others.*

What was the research question?

*Given their increased availability, do ED patients consider using alternative care options prior to reporting to the ED?*

What was the major finding of the study?

*Among ED patients, 14.1% met the avoidable visit criteria, providing an opportunity to improve resource allocation.*

How does this improve population health?

*As a safety net for medicine and society, EDs can become overburdened. Alternative care options for non-emergent cases may help alleviate the load on EDs, to focus on the sickest patients.*

## INTRODUCTION

Emergency departments (ED) have become a haven for patients seeking urgent medical attention. As required by federal law, EDs cannot refuse evaluation and emergency treatment, regardless of the patient’s ability to pay.^1^ A 1996 study revealed that 11.3% of ambulance transports were considered unnecessary, highlighting a positive correlation between these visits to the ED and limited transportation options.^2^ Given the increased availability of alternative transportation choices today compared with 1996, including public transportation and ride-sharing services, this correlation may have shifted. The current medical landscape also provides various alternatives for managing emergent medical conditions, including seeing primary care physicians (PCP), visiting urgent care centers (UCC), and using telehealth services.

The rise of alternative medical care options raises questions about their impact on reducing ED visits for conditions treatable through PCPs, UCCs, or telehealth services. Recent studies have examined why patients opt for the ED over other medical treatment facilities, citing factors such as limited access to or confidence in primary care, perceived urgency, convenience, recommendations from other physicians, friends, or family, and the belief that their condition necessitated resources provided by hospital-based emergency care.^3^ ^–^ ^11^

Despite the finding that 13.7–27.1% of all ED visits could be evaluated and treated at UCCs or retail clinics with lower cost, patients still frequently choose EDs for nonemergent care.^7^ ^,^ ^12^ Another common occurrence among patients visiting the ED with lower acuity conditions is unnecessary referral from a PCP or UCC.^13^ ^,^ ^14^ One study found that there were significantly more avoidable referrals from PCPs (13.9%) than UCCs (7.9%).^13^ Zitek et al found that 35.9% of the patients enrolled in their study who transferred from a UCC to the ED were considered an unnecessary transfer.^14^

Limited research has examined patient decision-making when choosing between the ED and UCC. A 2018 study highlighted patients’ uncertainty about what constituted urgent care, focusing on psychological factors rather than societal or physical determinants.^15^ Mukamel et al (2019) addressed these factors, emphasizing out-of-pocket costs and wait times for several medical conditions and care choices.^8^ Their findings revealed that lower out-of-pocket costs were prioritized over wait time for conditions lower in severity or acuity, whereas wait time gained importance for conditions perceived to be more urgent.^8^

The current study provides a comprehensive assessment of ED patients’ choices for care and explores the factors and obstacles impacting patients’ choices of a particular ED over a PCP, UCC, telehealth service, or another nearby ED. Additionally, we wanted to assess how avoidable some of these ED visits could be by examining patients’ perceptions of PCPs’, UCCs’, and telehealth services’ abilities to care for the medical conditions that caused them to seek care in the ED and to understand the selection patterns within this group. Understanding the location, size, clientele, and specialized care of the EDs may provide insight as to why individuals opt for one medical care option over another. A thorough examination of patients’ choices could offer valuable insights into enhancing the availability and accessibility of various medical care options for individuals with urgent conditions.

## METHODS

### Study Design

This study consisted of a multi-hospital survey and electronic health chart review. Research associates (RA) administered the survey to patients seeking care at three separate hospital EDs. Surveys were administered during normal business hours when most other care options would be open and available. Participants provided written consent at their bedside prior to completing the survey. The survey included general demographic questions and several questions regarding their decision to seek care in the specific ED in which they were approached, as opposed to using a PCP, UCC, telehealth service, or another local ED. We conducted a subsequent health chart review for diagnoses, tests/procedures, consults, and final disposition for each participating patient. The survey was developed from a previous study conducted locally,^2^ adapted to fit current standards and medical care options, and it was reviewed by a group of local emergency physicians.^13^ ^,^ ^16^ This study was approved by the institutional review board at the University at Buffalo.

### Setting

The survey was conducted at three local hospital sites located in a single county. Two are in the center of a metropolitan area and one in a city suburb (Table 1). Sites 2 and 3 are part of the same hospital system. The EDs at all three sites are staffed by the same physician group. The population of the local county is about 950,000 as of 2021 and includes the city with a population of about 277,000.

**Table 1.** Hospital site information and statistics.^17^ ^–^ ^20^

Hospitals	Hospital type	Beds	Location	Specialization	ED patients per year
Site 1	County	573	Urban	Full service, regional Level I trauma center	70,000
Site 2	Not for profit	484	Urban	Full service, regional stroke and STEMI center	64,000
Site 3	Not for profit	265	Suburban	Full service	50,000

*ED*, emergency department; *STEMI*, ST-Elevation Myocardial Infarction.

### Data Collection

Each RA was trained by the study coordinator at Sites 1 and 2 on the proper procedures for reviewing the consent form, administering the survey, and collecting the final data outcomes. All enrollments at Site 3 were done solely by the study coordinator. Enrollment for this study began on January 3, 2023, and concluded on May 1, 2023. Data collection took place at all three sites between 10 am – 10 pm, Sunday-Saturday. Subjects were included if they were at least 18 years old, read and spoke English, and had the capacity to provide consent to participate. The RAs at each hospital then consulted with the patient’s clinicians to determine whether the patient was able to give consent to participate in the survey. The RAs did not approach patients if they were altered, too sick to participate, mentally incapable, non-English speaking, sleeping, potentially infectious, receiving care, >89 years old, or reported by staff as being too agitated or upset to participate. Additionally, prisoners were not considered for this study as the location of their care is arranged without their input. If patients were unable to be approached for these reasons, they were recorded as ineligible.

After written consent was received, the survey was administered verbally, and every answer was recorded on an iPad using REDCap 10.3.3 (Research Electronic Data Capture Vanderbilt University, Nashville, TN) data management platform software hosted at University at Buffalo. All questions from the survey were asked to the patients as open-ended, but the RAs who asked the question would categorize the answer based on the survey options. The RAs were trained on how to categorize each response by the study coordinator. If the response did not fit any of the provided categories, it would be labeled as “other,” and the RA would describe the answer on REDCap via a blank text box.

After each participating patient was discharged, admitted, or transferred from the ED, RAs recorded their discharge diagnosis, any tests and procedures done, any specialists consulted, and the final disposition. We used this information to determine whether the patient’s visit to the ED could be categorized as avoidable. We defined an ED visit as avoidable if the patient did not have a high-acuity triage category of level 1 (resuscitation) or level 2 (emergent), was not admitted to the hospital or transferred, had no advanced imaging, had no specialist consultation while in the ED, and did not have a discharge diagnosis of chest pain or syncope. We defined advanced imaging as any imaging other than a radiograph (eg, computed tomography, magnetic resonance imaging, or ultrasound). These criteria were based on previous research coupled with a consensus from local emergency physicians to fit regional standards.^2^ ^,^ ^13^ ^,^ ^16^

### Analysis

We analyzed the data obtained from the surveys using SPSS Statistical Software v 27 (IBM Corporation, Armonk, NY) after it was exported from REDCap. Descriptive statistics were used to analyze the responses to the survey and present the data.

## RESULTS

Across the three hospitals, 52,246 patients reported to the ED during the study period. Of the 1,665 people considered for participation, 958 (57%) were approached and 590 (35.4%) consented to participate in the survey, resulting in a 1.1% study sample of the total patient population during the collection period (Table 2). Most participants were female (60.6%) and White (75.8%) (Table 3).

**Table 2.** Study sample representation of emergency department population. Emergency Severity Index.

	Study patients		Total ED patients during study period	
Hospitals	Patients surveyed	Average triage ESI score	ED patients seen	Average triage ESI score
Site 1	198	2.52	18,041	2.75
Site 2	197	2.75	18,122	2.85
Site 3	195	2.88	16,083	2.88
Total:	590	2.71	52,246	2.82

*ED*, emergency department; *ESI*, emergency severity index.

**Table 3.** Demographics of consented participants.

	Total (N = 590)
Gender	
Male	228 (38.5%)
Female	359 (60.6%)
Other	3 (0.5%)
Prefer not to answer	0 (0.0%)
Age	
Mean Years	51.15
SD	17.82
Race	
Black	113 (19.1%)
Asian/Pacific Islander	10 (1.7%)
White	449 (75.8%)
Native American	11 (1.9%)
Other	19 (3.2%)
Prefer not to answer	2 (0.3)
Hispanic/LatinX	
Yes	46 (7.8%)
No	544 (91.9%)
Highest level of education	
No high school	2 (0.3%)
Some high school	38 (6.4%)
High school graduate	153 (25.8%)
Some college	121 (20.4%)
Associate’s degree	78 (13.2%)
Bachelor’s degree	113 (19.1%)
Postgraduate degree	78 (13.2%)
Trade/technical training	7 (1.2%)
Other	0 (0.0%)
Type of health insurance	
Private	374 (63.4%)
Medicare	112 (19.0%)
Medicaid	88 (14.9%)
Uninsured	13 (2.2%)
Military	3 (0.5%)
Other	0 (0.0%)

The most common methods of transportation reported were having a family member or friend drive them (43.9%), followed by ambulance transport (28.3%) and driving themselves to the hospital (19.7%). Of those patients who had a family member or friend drive them, most of them described the reason as being too sick to drive themselves (67.2%). Of those patients who came in an ambulance, around half of them explained that they felt they needed immediate medical attention (48.5%). Additionally, most of those who arrived by ambulance stated they either called the ambulance themselves (32.9%), or a family member or friend called one for them (37.1%).

**Figure 1.**
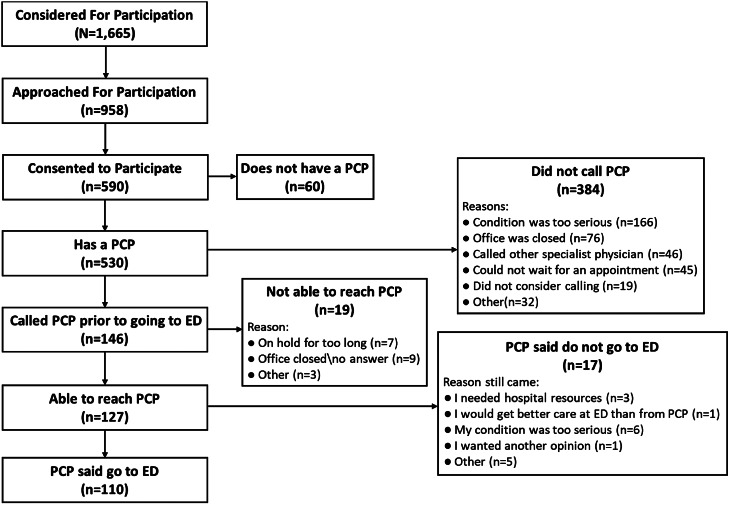
Description of involvement of primary care physician (PCP) in decision to go to the ED.

**Figure 2.**
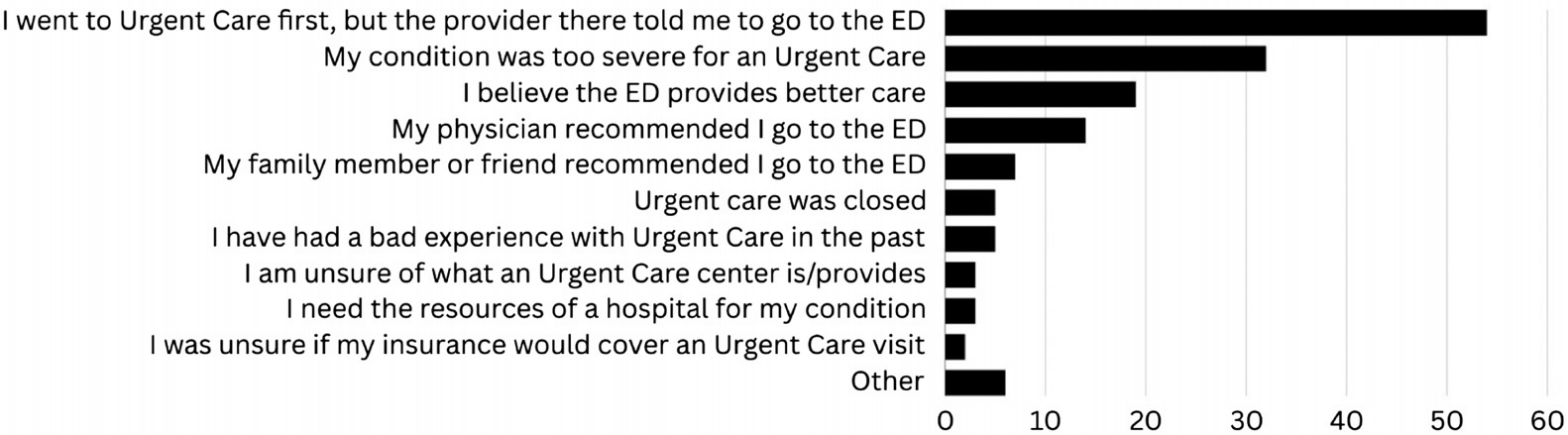
Reasons why patients chose the emergency department (ED) instead of urgent care center (UCC).

Of the 590 patients, 530 (89.8%) reported having a PCP. Only 146 (27.5%) of those 530 attempted to reach out to their PCP, with 127 (24.0%) making contact. Among those 127 patients who successfully contacted their PCP, 110 (86.6%) stated that their physician advised visiting the ED (Figure 1). Of those 110 patients, 75 (68.2%) had a triage category of 3 and 30 (27.3%) had a triage category of 2 (Table 4). Among the 152 (25.7%) patients who considered visiting a UCC, 135 (88.8%) had used a UCC in the past, 148 (97.4%) were aware of a UCC in their area, and 140 (92.1%) said they would consider using a UCC in the future. When asked why they chose the ED over a UCC, 54 (35.5%) patients reported they went to a UCC first, but the UCC clinician recommended they go to the ED. The second most common answer was the patient believed their condition was too serious for a UCC, 33 (22.4%) (Figure 2).

**Table 4.** Triage category compared to recommendation from primary care physician (N = 127).

	Triage Category				
	1: Resuscitation	2: Emergent	3: Urgent	4: Less urgent	5: Nonurgent
Doctor recommended going to ED (n = 110)	2 (1.8%)	30 (27.3%)	75 (68.2%)	3 (2.7%)	0 (0%)
Doctor did NOT recommend going to ED (n = 17)	0 (0%)	5 (29.4%)	11 (64.7%)	1 (5.9%)	0 (0%)

*PCP*, primary care physician; *ED*, emergency department.

Only 18 patients (3.1%) considered using telehealth services. Of those 18, 12 (66.7%) sought care through a telehealth service visit in the past, 16 (88.9%) would consider using telehealth services in the future, and 10 (55.6%) stated they believed they needed the resources of a hospital, which is why they chose the ED over a telehealth service visit.

The main reasons why patients chose their respective EDs over other local EDs included prior use of healthcare services at that hospital (23.9%), living near the ED (21.2%), and the belief that the hospital offered the specialized services they needed (18.1%). There were 190 (32.2%) patients with a triage category of 1 or 2. A total of 254 (43.1%) patients were admitted to the hospital, 325 (55.1%) had advanced imaging, and 150 (25.4%) had a specialist consultation. At discharge, 125 (21.2%) patients had a diagnosis of chest pain or syncope. Of the 590 patients surveyed, only 83 (14.1%) patients met our criteria for being an avoidable visit.

## DISCUSSION

Patients’ perceived seriousness of their condition was the most common reason for seeking care at the ED instead of alternative sources of care (Figures 1 and 2). Previous studies suggest that many people choose to take an ambulance because someone else called the ambulance for them or because of the perceived urgency or uncertainty about their medical conditions.^21^ ^,^ ^22^ This aligned with our findings because, of the 167 patients who presented to the ED by ambulance, 81 (48.5%) stated that they needed immediate medical assistance and 29 (17.4%) stated that they were too sick or in too much pain to drive themselves. Furthermore, of the patients who had a family member or friend drive them to the ED, the reason of being too sick to drive themselves was far more common than the other possible reasons (67.2%).

Of the 590 patients surveyed, 384 (65.1%) did not attempt to contact a PCP prior to going to the ED despite 530 (89.8%) reporting that they had a PCP. Previous research cites patients favoring the ED due to perceived urgency, limited access to PCPs, and the convenience of readily available tests in the ED.^6^ ^,^ ^11^ ^,^ ^23^ ^,^ ^24^ Another study found the primary reason patients chose the ED instead of a PCP was the perception of speed and convenience. However, this finding may be contradictory because of prolonged ED wait times that may occur with less acute conditions.^25^ Similar to Gorodetzer et al (2020), we found more than double the number of referrals from PCPs compared to UCCs (110 vs 54, respectively).^13^ Most patients who did not call their PCP’s office stated that they believed their condition was too urgent, which is comparable to previous studies.^5^ ^,^ ^11^ ^,^ ^24^ ^,^ ^26^

Additionally, for those patients who went to the ED against the advice of their PCP, 56.3% stated that they believed their condition was too serious or that they thought they needed the resources of a hospital for their condition (Figure 1), which aligns with previous research.^11^ Notably, of the patients who went to the ED despite not being advised to do so by their PCP, 29.4% had a triage acuity of 2 and 64.7% had a triage acuity of 3 (Table 4), indicating that some patients’ self-referral may have been more advantageous than if they had not chosen to visit the ED. Although this may identify an area for improvement for patients’ PCPs, it is difficult to accurately interpret situations because this study did not record whether patients called or physically visited their PCPs’ offices or to whom patients may have spoken to there.

Of the patients who considered visiting a UCC, most of them reported using a UCC in the past, knew of a UCC in their area, and said they would consider using a UCC in the future. This information contrasts the findings of Pope et al, suggesting that people in the United States might have more of a general awareness of what a UCC is and the services they may provide.^15^ In this study, patients who stated that they did not consider UCCs were not asked why. Adding this question to the survey may provide a better indication of the psychological, societal, and physical determinants as to why patients choose the ED over UCCs such as costs, wait times, and lack of understanding of UCC services, as previous literature suggests.^8^ ^,^ ^15^

To our knowledge, this is one of the first studies to investigate patient choices between ED and telehealth services. Although it has been shown that telehealth service use decreased ED volumes during the early days of the COVID-19 pandemic in 2020, the current study was conducted January–May 2023 and did not receive many responses (18, 3.1%) pertaining to the consideration of telehealth services in this population.^9^ Ten participants (55.6%) of the 18 who considered telehealth stated they chose the ED over telehealth services because of the perceived need for hospital resources, similar to the reasoning behind choosing the ED over PCP offices or UCCs. Furthermore, when this survey was conducted, not all health insurances policies covered telehealth services, potentially limiting their impact.

Understanding the significant decision-making behavior and future considerations for these patients is challenging, given that only 14.1% of the patients met our criteria for avoidable visits. Additionally, several studies have attempted to label a “non-urgent” ED visit in the past, each with different criteria, sample size, study design, and results.^8^ ^,^ ^12^ ^–^ ^14^ This complicates distinguishing between those who truly required ED care and those who might not have needed it, creating a convoluted and ambiguous process.

## LIMITATIONS

For privacy, surveys were conducted after patients were assigned and moved to an ED room. Patients who were treated in non-private areas such as fast track, hallway bed, or waiting room-adjacent areas were not included; these may represent a group with a greater ratio of avoidable visits. Additionally, RAs were unable interview patients who presented to the ED and left before receiving treatment.

The requirement for RAs to review a consent form and obtain a signature from the patient may have resulted in reluctance or hesitation for participation and subsequent declination to participate from 109 patients for multiple reasons. First, in their review of the consent form, RAs were required to explain that the study team would be obtaining basic information from the patient’s health record after the patient completed the survey, which may have been perceived as a potential breach of confidentiality. Next, reviewing the consent document took approximately four minutes, which could have been enough time for the prospective subjects to lose interest, potentially feel too ill to participate, or for a clinician to intervene during the enrollment process. Lastly, the regulatory requirement of obtaining written consent may have decreased the potential number of patients that could have been enrolled in this study and may have introduced bias into our findings.

Discussing and answering questions about their ED visits may be an emotional or sensitive topic for patients. Although the RAs were trained to ask the questions in a non-judgmental and welcoming tone, some patients may have been disinclined to provide honest or complete responses. Additionally, going to the ED for some may be considered a traumatic experience, regardless of triage acuity, which may have reduced willingness to participate. Finally, patients who were too sick, intoxicated, or incapacitated were not approached to participate due to their condition. These patients were presumed to be an unavoidable ED visit; thus, omitting their data may impacted the results.

Future studies should focus on including rural hospitals compared to suburban and urban hospitals. Inclusion of a rural setting may contribute more data surrounding patients’ use of telehealth services. Additionally, including those patients seen in fast track or other lower acuity areas would provide more information on avoidable ED visits. Previous studies also found that there is a high number of avoidable pediatric patient visits to the ED.^14^ Incorporating pediatric patients and the decision-making of their accompanying adult(s) in future work could also shed light on how people decide where to go for their emergency care.

## CONCLUSION

Per our findings, individuals primarily rely on their perception of the severity of their condition when making decisions about seeking emergency care. While several patients contemplated alternative options such as scheduling a visit to a PCP’s office, visiting a UCC, or accessing healthcare through telemedicine services, the uncertainty surrounding their medical condition, recommendations from other healthcare professionals, and the perceived quality of care significantly influenced their choice in directing them to the ED. Non-emergent patients report to the ED for many reasons including a discrepancy in both understanding the severity of symptoms and determining the most suitable place to seek medical care.
